# *KIR* and *HLA* Loci Are Associated with Hepatocellular Carcinoma Development in Patients with Hepatitis B Virus Infection: A Case-Control Study

**DOI:** 10.1371/journal.pone.0025682

**Published:** 2011-10-05

**Authors:** Ning Pan, Wei Jiang, Hang Sun, Fengqin Miao, Jie Qiu, Hui Jin, Jinhuan Xu, Qian Shi, Wei Xie, Jianqiong Zhang

**Affiliations:** 1 Department of Immunology and pathogen biology, Southeast University Medical School, Nanjing, Jiangsu Province, China; 2 Key Laboratory of Developmental Genes and Human Disease, Ministry of Education, Institute of Life Science, Southeast University, Nanjing, Jiangsu Province, China; 3 Department of Medical Genetics, Southeast University Medical School, Nanjing, Jiangsu Province, China; 4 Department of Epidemiology, School of Public Health, Southeast University, Nanjing, Jiangsu Province, China; 5 The Second Affiliated Hospital of Southeast University, Nanjing, Jiangsu Province, China; Karolinska Institute, Sweden

## Abstract

**Background:**

Natural killer (NK) cells activation has been reported to contribute to inflammation and liver injury during hepatitis B virus (HBV) infection both in transgenic mice and in patients. However, the role of NK cells in the process of HBV-associated hepatocellular carcinoma (HCC) development has not been addressed. *Killer cell immunoglobulin-like receptors (KIRs*) are involved in regulating NK cell activation through recognition of specific *human leukocyte antigen* (*HLA*) class I allotypes.

**Methodology/Principal Findings:**

To investigate whether *KIR* and *HLA* genes could influence the risk of HBV-associated HCC development, 144 HBV-infected patients with HCC and 189 well-matched HBV infectors with chronic hepatitis or cirrhosis as non-HCC controls were enrolled in this study. The presence of 12 loci of *KIR* was detected individually. *HLA-A*, *-B*, *-C* loci were genotyped with high-resolution. *HLA-C group 1* homozygote (OR = 2.02; *p = *0.005), *HLA-Bw4-80I* (OR = 2.67; *p = *2.0E-04) and combination of full-length form and 22 bp-deleted form of *KIR2DS4 (KIR2DS4/1D)* (OR = 1.89; *p = *0.017) were found associated with HCC incidence. When the combined effects of these three genetic factors were evaluated, more risk factors were observed correlating with higher odds ratios for HCC incidence (*P* trend = 7.4E-05). Because all the risk factors we found have been reported to result in high NK cell functional potential by previous studies, our observations suggest that NK cell activation may contribute to HBV-associated HCC development.

**Conclusions/Significance:**

In conclusion, this study has identified significant associations that suggest an important role for NK cells in HCC incidence in HBV-infected patients. Our study is useful for HCC surveillance and has implications for novel personalized therapy strategy development aiming at HCC prevention in HBV-infected patients.

## Introduction

Hepatocellular carcinoma (HCC) is the fifth most common cancer worldwide and the third most common cause of cancer mortality. Globally, Hepatitis B virus (HBV) is the most frequent underlying cause of HCC. In hyperendemic areas such as China and Africa, chronic HBV infection contributes to at least 80% of cases of HCC [1]. There is intense interest in cellular and molecular mechanisms underlying HBV-associated HCC incidence. However, due to the long duration (usually more than three decades) from HBV infection to HCC incidence and the complexity of carcinogenesis, mechanism underlies the HCC development in hepatitis B patients is poorly understood.

Persistent inflammation was recognized to function as a driving force in the journey to HCC as well as in many other cancers [2], [3]. Although accumulating reports support that NK cell activation contribute to inflammation and liver injury during HBV infection both in HBV transgenic mice and in HBV infected patients [4]–[7], the role of NK cells in the process of HBV-associated HCC development has not been addressed.

The activation of NK cells is dependent on the equilibrium between the inhibitory and activating receptors, among which *immunoglobulin-like receptors (KIRs)* are by far the most polymorphic. Through recognition of specific *human leukocyte antigen* (*HLA*) class I allotypes ligands, *KIR* contributes to the array of receptor–ligand interactions that determine NK cell response to its target [8], [9]. *KIR* function can be predicted from the length of the cytoplasmic domain, where long (L) receptors (*KIR2DL/KIR3DL*) are generally inhibitory and all short (S) receptors (*KIR2DS/KIR3DS*) are activating. Two groups of human KIR haplotypes are defined based on gene content, termed haplotype A and haplotype B [8]. The A haplotype carries a fixed organization of seven genes with only one activating gene (*KIR2DS4*), while the B haplotype contains varying combinations of *2DS1*, *2DS2*, *2DS3*, *2DS4*, *2DS5* and *3DS1*. *KIR1D* is the mutant form of KIR2DS4 with a 22 bp deletion in exon 5 which causes a frame shift, resulting in a truncated *KIR2DS4* protein that would be secreted due to the loss of the transmembrane/cytoplasmic domains. The function of *1D* is unclear [10], [11].

As one of the ligands for *KIR*, *HLA-C* molecules are classified as either *HLA-C group 1* (*HLA-C1*) or *HLA-C group 2* (*HLA-C2*) on the basis of dimorphisms at position 80. *KIR2DL1* recognizes *HLA-C2* molecules that are characterized by lysine at position 80, whereas *KIR2DL2* and *KIR2DL3* prefer *HLA-C1* molecules contain asparagine at this position. *HLA-Bw4* allotypes are known to bind *KIR3DL1* [12], [13]. The remaining *HLA-B* allotypes are typified by a *Bw6* motif, which is not recognized as *KIR* ligand. *HLA-Bw4*-containing allotypes with isoluecine at position 80 (*Bw4-80I*) generally bind *KIR3DL1* with higher affinity than allotypes with threonine at this position (*Bw4-80T*) do [8], [14]. The polymorphisms of *KIR* and *HLA* could influence the differences in human natural killer cell responsiveness and potency. *HLA-C1/KIR2DL3* inhibited-NK cells are recognized with higher function potential than those inhibited by *HLA-C2/KIR2DL*1 [9], [15], [16]. Individuals carrying *Bw4* have more potent NK cells than individual without *Bw4* do, because *KIR3DL1+* NK subset in *Bw4+* subjects exhibits higher level of cytokine productivity and cytotoxicity in response to various stimuli [17]–[19]. The ligands of activating *KIRs* have not been determined.

Three virus-associated carcinomas have been reported to be associated with *KIR* and *HLA* genes. However, opposite roles of NK cell activation were suggested in etiology of these viral associated cancers. In cervical neoplasia, which is caused by human papilloma virus, and in nasopharyngeal carcinoma, which is associated with EBV infection, *KIRs* or *KIR/HLA* compound genotypes expected to result in an activating phenotype increase the risk of carcinoma development, suggesting NK cell activation may contribute to these virus-associated carcinomas [20], [21]. On the contrary, a study on HCV-associated HCC demonstrates that a combination which conveys activating signal protected against the development of HCC [22].

To investigate the influence of *KIR* and *HLA* genes on the risk of HBV-associated HCC development, a case-control study was conducted in HBV-infected patients with HCC and well-matched HBV-infected patients without HCC as non-HCC controls. Three genetic factors were found associated with HBV-associated HCC incidence. Interestingly, all of these genetic risk factors have been reported to result in high NK cell function potential by previous studies, suggesting that NK cell activation might take part in the process of HBV-related HCC development.

## Materials and Methods

### Ethics Statement

The protocol was approved by the ethics committee of the Second Affiliated Hospital of Southeast University, and all patients provided written, informed consent before enrollment.

### Subjects

From 2005 to 2007, 333 patients with persistent HBV infection were enrolled from the Second Affiliated Hospital of Southeast University according to a case-control study design. Among 333 patients, 144 were diagnosed as primary HCC with liver cirrhosis (defined as 1 or more tumoral nodules in a cirrhotic liver), 88 patients with liver cirrhosis and 101 patients with chronic hepatitis. All diagnoses of HCC and liver cirrhosis were defined by clinical and biological criteria and confirmed by image technologies (computed tomography and echography).

To exclude other host risk confounders involved in HCC development, all the patients selected here were free of other hepatic virus co-infection, alcohol consumption, and with no sign of autoimmune disease. HCC patients whose ages range from 20 to 65 were selected for this study. Age distribution and gender composition were matched in the three diagnostic groups. All patients were members of Han population and lived in the same geographical area. *KIR* genotyping was accomplished in each of the subject. Because *HLA* typing could not be accomplished for some cases (because of lack of DNA), final analysis of combinatorial *KIR/HLA-C/HLA-B* effects included 124 cancer cases and 169 controls (94 with chronic hepatitis and 75 with cirrhosis).

### *KIR* genotyping

PCR amplification was performed with primers specific for each locus for the presence or absence of the following activating KIR genes: *2DS1*, *2DS2*, *2DS3*, *2DS4* (full-length form), *1D* (the 22 bp-deletion mutant form of *2DS4*), *2DS5*, and *3DS1*; and inhibitory KIR genes: *2DL1, 2DL2, 2DL3, 2DL5, and 3DL1*. Internal positive control primers for fragment of framework gene *KIR2DL4* were also included in each PCR reaction. All primer sequences and amplification conditions were as described in previous report [23].

### *HLA* genotyping

A routine sequence-based typing method was performed. Genomic DNA was extracted from peripheral blood mononuclear cells by a standard salting-out method and a region including exon 2 and 3 of *HLA-A*, *HLA-B* and *HLA-C* loci respectively was amplified using locus-specific primers [24]. Sequencing reactions were performed using the BigDye® Terminator v3.1 Cycle Sequencing Ready Reaction Kit (Applied Biosystems) and exons 2 and 3 of each locus were sequenced in both forward and reverse directions using a 3730XL DNA Analyzer (Applied Biosystems, Foster City, CA). The sequences were then analyzed using online dbMHC SBT typing tool [25].

### Novel *HLA* class I alleles

Two novel HLA class I alleles (HLA-A*9216 and HLA-Cw*0134) were identified in this study [26], [27]. Nucleotide sequence of all the new alleles have been submitted to the GenBank nucleotide sequence database and is available under the accession number EF468681 (for HLA-A*9216) and GQ365731 (for HLA-Cw*0134).

### Statistical methods

Allelic frequencies were initially determined for HCC and control groups, and the significances of genotypic and allelic associations were determined either by Pearson's x^2^ test or by Fisher's exact test (when there were less than five subjects in a cell). The level of significance of each test was adjusted for multiple testing using the Bonferroni's correction. Multivariate Logistic regressions and Spearman correlation tests were conducted for some analyses as described. *HLA-C* genotype frequencies were checked for the Hardy-Weinberg equilibrium using Pearson's x^2^ test. Odds ratios (ORs) and 95% confidence intervals (CIs) were calculated to determine the magnitude and statistical significance of associations. All tests of statistical significance were 2-sided. Statistical analyses were performed using SPSS software (Version 11.0 Chicago IL, SPSS Inc.).

## Results

Male gender, older age (or longer duration of infection), cirrhosis, alcohol consumption, and co-infection with HCV or HDV are acknowledged HCC host risk factors involved in HCC development [1]. To exclude these risk confounders, all the patients in this study were free of other hepatic virus co-infection, alcohol consumption, and with no sign of autoimmune disease. HCC patients included in this study all had liver cirrhosis. Patients with chronic hepatitis and cirrhosis were matched with HCC patients in age and gender. Age distribution and gender composition showed no statistical difference both between HCC and non-HCC groups and among three diagnostic groups (Table 1).

**Table 1:** Demographic and clinical features of HBV-infected patients included in the present study.

Variable	Non-HCC (n = 189)		HCC(n = 144)	*p*-value	
	Hepatitis (n = 101)	Cirrhosis (n = 88)		Among 3 diagnostic groups	HCC vs non-HCC
Age (Mean ± SD)	43.6±9.7	45.3±8.2	45.3±7.2	0.20	0.33
Gender (Male %)	79 (78.2)	65 (73.9)	120 (83.3)	0.21	0.11
Other Hepatic Virus Co-infection*	0	0	0	-	-
Alcohol Consumption	0	0	0	-	-

*Patient sera were tested for hepatitis A virus IgM, hepatitis C virus antibody, hepatitis D virus antigen, hepatitis D virus antibody, and hepatitis E virus IgM.

### Combination of *KIR2DS4* and *1D* was associated with disease progression towards HCC development

The frequencies of *KIR* genes were analyzed first. *KIR2DL1* (98.5%), *KIR2DL3* (98.8%) and *KIR3DL1* (95.3%) were present in nearly all individuals. The phenotype frequencies of other KIR loci varied from 16.8% to 79.0% in the whole population of this study. However, no significant difference on any given locus was found between HCC and control groups (table S1).

Among the *KIR* genes we tested, only *KIR2DS4 (full-length form)* and *1D (22bp-deleted form of KIR2DS4)* have identical outer membrane sequences. When we examined the effect of the combination of activating *KIR2DS4* and *1D*, we found that the frequency of *KIR2DS4/1D* was increased in HCC patients (16.9% in non-HCC group, 27.8% in HCC group, *p* = 0.017, OR = 1.89, 95%CI = 1.14–3.20). Multivariate regression analysis suggested that this susceptive effect of *KIR2DS4/1D* (*p* = 0.008) on HCC incidence was independent of the influence of other activating *KIRs*, when *KIR2DS1* (*p* = 0.49), *2DS2* (*p* = 0.22), *2DS3* (*p* = 0.09), *2DS5* (*p* = 0.13) and *3DS1* (*p* = 0.66) were included as covariates. Furthermore, when distribution of *KIR2DS4/1D* was examined among chronic hepatitis, cirrhosis and HCC diagnostic groups, *KIR2DS4/1D* frequencies in hepatitis, cirrhosis and HCC patients were observed to be increased in turn significantly (P trend = 0.004, determined by Spearman correlation test), and this trend is dependent on both presence of *KIR2DS4* and *1D* (table 2). This observation indicated that the *KIR2DS4/1D* was linked with disease progression from hepatitis via cirrhosis to HCC development.

**Table 2:** Associations between *KIR2DS4/1D* with disease progression towards HCC development.

*KIR*	Hepatitis n = 101	Cirrhosis n = 88	HCC n = 144	*P* trend
	n (%)	n (%)	n (%)	
*KIR2DS4*	76 (74.5)	69 (77.5)	121 (81.8)	0.17
*KIR1D*	26 (25.7)	30 (33.3)	53 (36.8)	0.19
*KIR2DS4−/1D−*	11 (10.9)	10 (11.4)	13 (9.0)	0.60
*KIR2DS4−/1D+*	14 (13.9)	9 (10.2)	13 (9.0)	0.25
*KIR2DS4+/1D−*	64 (63.4)	49 (55.7)	78 (54.2)	0.17
*KIR2DS4+/1D+*	12 (11.9)	20 (22.7)	40 (27.8)	0.004

### Higher *HLA-C1* copy number was associated with increased disease progression to HCC

To test the possibility that the *KIR* genes are involved in risk of HBV-associated HCC incidence, we genotyped *HLA-A*, *-B* and *–C* loci, and grouped them according to *KIR* ligand.

**Figure 1:**
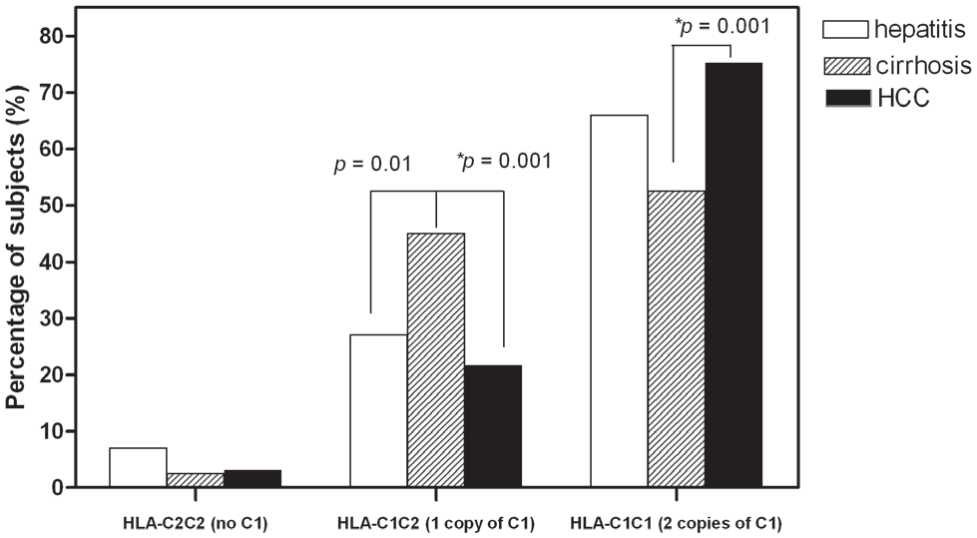
Effect of copy number of *HLA-C1* on disease progression to HCC development. The frequency of *HLA-C1C2* (1 copy of *HLA-C1*) was increased significantly in cirrhosis patients compared to that in HCC patients (cirrhosis vs HCC: OR = 2.95, *p* = 0.001). This increase in cirrhosis patients did not reach statistical significance level after correction when compared to that in hepatitis patients (cirrhosis vs hepatitis: OR = 2.21, *p* = 0.01). *HLA-C1C1* (2 copies of *HLA-C1*) was found more frequently in HCC patients than that in liver cirrhosis patients (HCC vs cirrhosis OR = 0.34, *p* = 0.001) (table S1). * Of significant difference (*p*<0.006) after correction.

As *KIR* ligand, *HLA-C* molecules are classified as either *HLA-C1* or *HLA-C2* on the basis of dimorphisms at position 80. *HLA-C* genotype frequencies were consistent with Hardy-Weinberg equilibrium (p = 0.46). We found that genotype *HLA-C1C1* was associated with HCC incidence (*p* = 0.005, OR = 2.02, table 3). Moreover, when the frequencies of *HLA-C* genotypes were analyzed among chronic hepatitis, cirrhosis and HCC diagnostic groups, one copy of *HLA-C1* (*HLA-C1C2*) was found associated with liver cirrhosis (vs chronic hepatitis; OR = 2.21; *p* = 0.01; vs HCC; OR = 2.95; *p* = 0.001), two copies of *HLA-C1* (*HLA-C1C1*) were found more frequently in HCC patients than that in the patients with liver cirrhosis (HCC vs cirrhosis OR = 0.34, *p* = 0.001) (table S2 and figure 1). So, higher *HLA-C1* copy number was found to be associated with increased disease progression to HCC.

**Table 3:** Associations between *HLA-C* ligands for *KIR* with HCC.

*HLA-C* genotypes	Non-HCC n = 180	HCC n = 129	*p*-value	OR (95% CI)
	n (%)	n (%)		
*HLA-C1C1*	108 (60.0)	97 (75.2)	0.005*	2.02 (1.23 to 3.33)
*HLA-C1C2*	63 (35.0)	28 (21.7)	0.011	0.58 (0.31 to 0.87)
*HLA-C2C2*	9 (5.0)	4 (3.1)	0.41†	0.61 (0.18 to 2.02)

*Of significant difference (*p*<0.017) after correction.

† determined by Fisher's exact test.

### *HLA-Bw4-80I* increased the risk of HCC occurrence

We then compared the distribution of the *HLA-B* ligand for *KIR* between HCC and non-HCC patients. The increase of *HLA-Bw4* frequency in HCC patients did not reach statistical significant level after correction (*p* = 0.028, OR = 1.65, table 4). The *HLA-Bw6* allele which was not the ligand for *KIR* had the same distribution in HCC and non-HCC groups (*p* = 0.46, OR = 0.80, table 4). *HLA-Bw4-80I* was recognized to bind *KIR3DL1* with higher affinity than *Bw4-80T* [8], [14], so we analyzed the frequencies of *Bw4*-containing allotypes based on their dimorphism at position 80. *Bw4-80I* allele was found to increase the risk of HCC development significantly (*p* = 0.0002, OR = 2.67), while no association was found in *Bw4-80T* allele (*p* = 0.22, OR = 0.74, table 4) .Within non-HCC controls, the distribution of *Bw4-80I* was comparable in chronic hepatitis patients to that in cirrhosis patients (19.2% in chronic hepatitis patients and 13.6% in cirrhosis patients). Multiple-variable logistic regression analysis showed the persistence of the susceptive effect of *Bw4-80I* (*p* = 0.001), when *Bw4-80T* (*p* = 0.57) and *Bw6* (*p* = 0.83) were included as covariates. No interactive effect between *HLA-Bw4-80I* and *KIR3DL1* was observed probably due to the nearly 100% presence of *KIR3DL1* both in HCC and in non-HCC groups (table S1).

**Table 4:** Associations between *HLA-B* ligands for *KIR* with HCC.

*HLA-B* ligands for *KIR*	Non-HCC n = 181	HCC n = 138	*p*-value	OR (95% CI)
	n (%)	n (%)		
*Bw4*	86 (47.8)	83 (60.1)	0.028	1.65 (1.58 to 4.51)
*Bw6*	153 (85.0)	113 (81.9)	0.46	0.80 (0.44 to 1.45)
*Bw4-80I*	30 (16.7)	48 (34.8)	0.0002*	2.67 (1.52 to 4.52)
*Bw4-80T*	64 (35.6)	40 (29.0)	0.22	0.74 (0.46 to 1.19)

*Of significant difference (*p*<0.013) after correction.

Although some *HLA-A* alleles were reported as putative ligands for *KI*R [28], [29], no association with HCC on any *HLA-A* allotypes was found (data not shown).

### Combined effects of *HLA-C1C1*, *HLA-Bw4-80I* and *KIR2DS4/1D* on HCC development

The combined effects of three genetic risk factors we found on HCC development were then examined. An at least additive effect on HCC development between *HLA-C1C1* and *Bw4-80I* was observed (*Bw4-80I^−^/HLA-C1C1^+^*, OR = 2.00; *Bw4-80I ^+^/HLA-C1C1^−^*, OR = 1.89; *Bw4-80I ^+^/HLA-C1C1^+^*, OR = 4.99, table S3), which opposed to a single susceptive locus that was simply marked by a second locus through linkage disequilibrium. Results of multivariate regression analysis confirmed that *Bw4-80I* (*p* = 0.001) and *HLA-C1C1* (*p* = 0.007) both contributed to HCC incidence.

**Figure 2:**
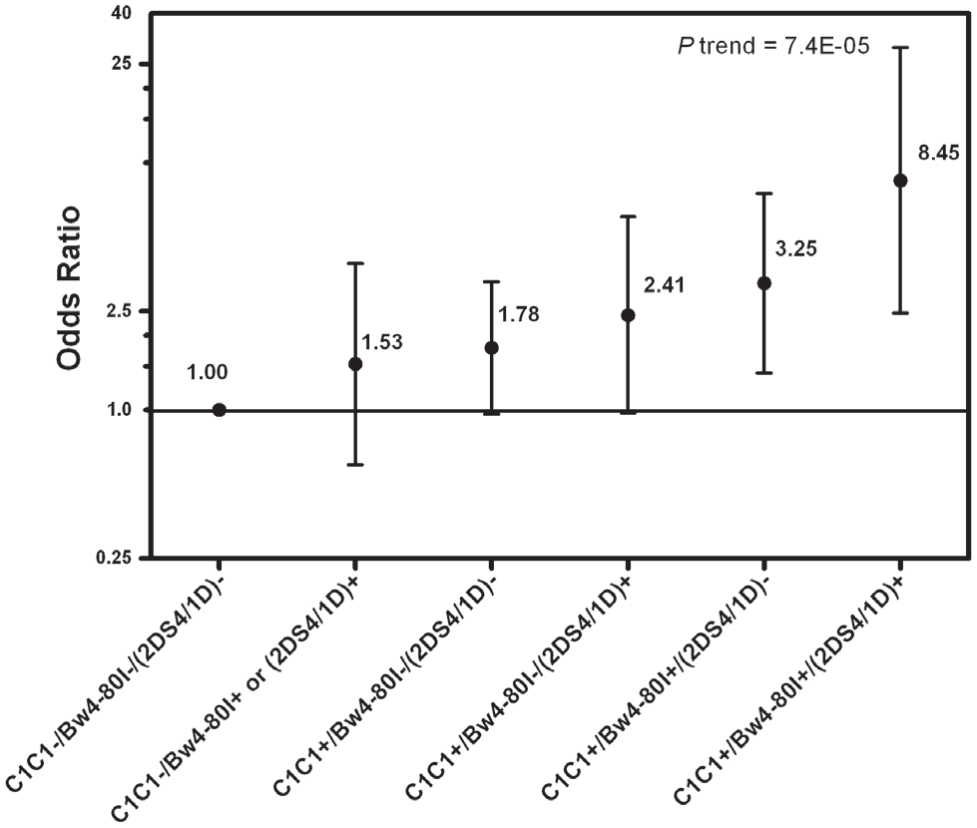
Combined effect *HLA-C1C1/Bw4-80/(KIR2DS4/1D)* on HCC occurrence. ORs (circle) and 95% confidence intervals (dashes) are shown for *C1C1/Bw4-80/(KIR2DS4/1D)* separately. The referent groups, *C1C1-/Bw4-80-/(2DS4/1D)-* are those with which all other genotypically defined groups are compared, and the OR for referent group is set at 1. We listed the various genotypes separately and ordered by increasing ORs as a means to compare ORs of individual genotypes (table S3). Group *C1C1-/Bw4-80+/(2DS4/1D)+* was not included because only 2 subjects in HCC group and 1 subject in control group carried this genotype.

The combined effects among *HLA-C1C1*, *Bw4-80I* and *KIR2DS4/1D* were then evaluated. A significant test for trend determined by Spearman correlation test was observed for ORs when more risk factors were present (P trend = 7.4E-05): (a) absence of *Bw4-80I*, *KIR2DS4/1D* and *HLA-C1C1*; (b) presence of *KIR2DS4/1D* or *Bw4-80I*, absence of *HLA-C1C1*; (c) absence of *KIR2DS4/1D* and *Bw4-80I*, presence of *HLA-C1C1*; (d) absence of *Bw4-80I*, presence of *KIR2DS4/1D* and *HLA-C1C1*; (e) absence of *KIR2DS4/1D*, presence of *Bw4-80I* and *HLA-C1C1*; (f) presence of *Bw4-80I*, *KIR2DS4/1D* and *HLA-C1C1* (table S3, figure 2). The data suggest that *HLA-C1C1* may be the major risk factor among *Bw4-80I*, *HLA-C1C1* and *KIR2DS4/1D*. The presence of both *Bw4-80I* and *KIR2DS4/1D* may have stronger susceptive effect than having only one of these risk genes when *HLA-C1C1* is present.

## Discussion

To our knowledge, this is the first report to establish a close correlation between *KIR*, *HLA* loci and HBV-associated HCC development. Interestingly, all the risk factors we identified here (*HLA-C1C1*, *HLA-Bw4-80I* and *KIR2DS4/1D*) had been reported to result in high NK cell functional potential by previous studies.

For *HLA-C1C1*, direct binding assay and disease association studies all suggest that *KIR2DL3/HLA-C1* interaction results in higher NK cell function level than other *HLA-C/KIR* interactions [16]. For example, *KIR2DL3/HLA-C1* has been shown to be associated with resolution of hepatitis C virus [30]. Functional analyses also demonstrate that NK cells in *HLA-C1C1* subjects exhibit more rapid and stronger antiviral response than those in *HLA-C2C2* subjects due to different responses of HLA-C-inhibited NK subsets [9]. In our study, *KIR2DL3* (98.8%) were present in nearly all individuals. Therefore, the presence or absence of its *HLA* ligands, *HLA-C1*, determines the existence of *KIR2DL3/HLA-C1* interaction in any given individual. Moreover, our results showed that more copies of *HLA-C1* alleles, which resulted in inherently more potent NK cells, were associated with disease progression towards HCC (one copy associated with cirrhosis; two copies associated with HCC) in HBV-infected patients (table S2 and figure 1), suggesting that NK cell activation may play a role in HCC development.

The frequency of *KIR* ligand for *HLA-Bw4-80I, KIR3DL1*, was also near 100% in our study (table S1), so the presence of *HLA-Bw4-80I* also determines the presence of *HLA-Bw4-80I/KIR3DL1* interaction in any given individual. Recent functional studies carried out by three independent research groups reveal that individuals carrying *KIR3DL1/HLA-Bw4* receptor-ligand pairs are of higher NK cell functional potential than *Bw6/Bw6* individuals do, because *KIR3DL1*+ NK subset in *HLA-Bw4+* individuals are more potent than its counterpart in *Bw4*- individuals [17]–[19]. This is consistent with NK cell licensing [31], [32]. According to licensing theory, the recognition of self *HLA* class I by inhibitory *KIR* is involved in the calibration of NK cell effector capacities during a developmental stage, only NK cell subsets with inhibitory *KIR* that has been recognized by self-*HLA* (*KIR3DL1* recognized by *Bw4* in this case) during maturation are of full functional competence. Moreover, findings in transgenic mice support that higher ligand-receptor interaction may affect NK licensing and result in higher functional potential [33]. In the present study, *HLA-Bw4-80I* allele, which binds *KIR3DL1* with higher affinity than the whole *HLA-Bw4* allele, was observed to increase the risk of HCC incidence, indicating that NK cell activation might be involved in HBV-associated HCC development, which is consistent with our findings on *HLA-C* locus.

The ligands of activating *KIRs* have not been determined. Few data are available regarding ligands for *KIR2DS4* and the physiological role of *KIR2DS4* remains unknown. Katz et al. report that *KIR2DS4* is able to interact with a non-*class I MHC* protein expressed on melanoma cell lines and on a primary melanoma to enhance NK cell activation [34]. However, they fail to identify the ligand. We observe that the frequency of *KIR2DS4* is higher in HCC patients than in non-HCC controls, although it does not reach statistical level (84.0% in HCC patients compared to 76.7% in controls, *p* = 0.099). Whether *KIR2DS4* could bind protein on HBV-infected or transformed cells needs further investigation. *KIR1D* has a 22 bp deletion in exon 5 which results in a truncated *KIR2DS4* protein that would be secreted due to the loss of the transmembrane/cytoplasmic domains. The function of *1D* is unknown. Our results raise the possibility that *KIR1D* molecule might be functional through interaction with the ligand of *KIR2DS4*. However, the mechanism underlying this genetic association remains unknown due to the lack of information of ligand for *KIR2DS4* and *1D*.

In spite of conflicting reports, some activating *KIRs* are reported to be able to bind classical *HLA* molecules with low affinity, such as *KIR2DS1/2DS2-HLA-C1*, *KIR2DS3-HLA-C2*, and *KIR3DS1-HLA-Bw4*. All of these putative receptor-ligand combinations were analyzed in our study; however no significant result was observed (data not shown).

Because most cases of HCC are detected lately and usually fatal within a few months of diagnosis. Hepatitis B treatment and surveillance aiming at HCC prevention is of great importance. However, mechanism underlies the HCC development in hepatitis B patients is poorly understood, which impedes the HCC prevention in hepatitis B patients. Our results indicate that innate immune response mediated by NK cell might contribute to intrahepatic inflammation process initiated by long-term HBV infection and eventually increase the risk of HCC development.

NK cells are highly enriched in the liver and comprise the dominant intrahepatic lymphocyte population, yet their role in HBV-related liver damage has not been well defined. The CD56^dim^ subset expresses the majority of NK cell perforin and granzyme, but hepatocytes are relatively resistant to these classical cytolytic effector molecules [35]. The CD56^bright^ subset of NK cells, noted to be selectively enriched in the periphery during hepatic flares and preferentially enriched and activated in the liver, is a potent source of cytokines such as interferon (IFN)-gamma. In HBV transgenic mice, NK cells are reported to mediate the over-sensitive liver injury in an IFN-gamma-dependent manner [5]. Through induction of chemokines, adhesive molecules, and proapoptotic proteins, IFN-gamma plays an important role in the development of hepatic inflammation and liver injury. Higher expressions of IFN-gamma receptor on hepatocytes and stronger IFN-gamma signaling pathway are also observed which might account for the hypersensitivity to IFN-gamma induced hepatocyte injury. Additionally, NKG2D recognition of hepatocytes by NK cells is also reported to play a critical role in oversensitive liver injury in HBV transgenic mice [7]. In patients with chronic HBV infection, activated NK cells are found enriched in the liver during flares of liver inflammation, and contribute to liver inflammation by tumor necrosis factor-related apoptosis-inducing ligand (TRAIL)-mediated death of hepatocytes. The authors also demonstrate that this pathway could be further enhanced by cytokines such as IFN-alpha and IFN-alpha/IL-8 combination, suggesting that this NK cell-mediated liver injury can be switched on by cytokines produced during active HBV infection [4]. Our findings suggest that these pathways induced by NK cells may be also involved in HCC development as in hepatic flare incidence. Further studies on the role of NK cells during the whole course of HBV-associated HCC development are justified in order to reduce the intensity of the inflammatory response and risk of subsequent HCC among hepatitis B patients.

IFN-alpha therapy has been used for decades, and has been proven to be effective in chronic hepatitis treatment when short-term responses, such as alanine aminotransferase level and presence of hepatitis B e antigen are measured. Although many studies have been conducted, the long term effects of interferon therapy, such as prevention of incidence of cirrhosis and HCC, are still under disputation. A recent systematic review for a National Institutes of Health Consensus Development Conference concludes that evidence is insufficient to assess treatment effect on long-time clinical outcomes [36]. IFN is a potent NK cell activator both in vivo and in vitro. Dunn C. and colleagues observe a temporal correlation between flares of liver inflammation and fluctuations in IFN-alpha in patients with chronic HBV infection. They also report that IFN-alpha concentrations found in patients are capable of activating NK cells to induce TRAIL-mediated hepatocyte death in vitro [4]. Our observations indicate that over-activation of NK cells might facilitate HBV-associated HCC development, suggesting that the host genetic background of *KIR* and *HLA* loci should probably be taken into consideration when IFN therapy is applied in hepatitis B patients.

Longer duration of HBV infection is one of the important risk factors involved in HCC development. However, because HBV infectors can be asymptomatic for a few to more than 30 years [37], it is almost impossible to determine the duration of HBV infection for most HBV-related disease patients in China. It is reported by WHO that in WHO Western Pacific Region, persistent HBV infection mainly results from either vertical transmission at birth or horizontal transmission in children under 5 years of age [38]. A nationwide survey conducted in 1979–1980 in China, for example, reported an hepatitis B surface antigen seroprevalence of 8.9% in 1-4-year-olds, and changed little thereafter [39]. Therefore, age-matching design not only excluded one of the major risk confounders of HCC occurrence, but also made all three diagnostic groups comparable in terms of approximate duration of HBV infection. Although the design of age and sex-matching as well as other entry criteria (such as free of other hepatic virus infection and alcohol consumption) restricted our patient selection and led to relative small sample size, the strict-match design enhanced comparability of our case-control study and made our results more reliable.

A recent genome-wide association study (GWAS) identified 1p36.22 as a new susceptibility locus for HBV-related HCC [40]. In this study, Zhang and colleagues genotyped 440,794 SNPs in HCC patients and non-HCC controls lived in Guangxi province. Then they examined the top 45 significantly associated SNPs detected by GWAS in other independent samples and confirmed one SNP. No SNP within *HLA* or *KIR* region was found associated with HCC incidence in Zhang's study. We do not think it is in conflict with results of our observation. One of major reasons is that GWAS which is based on testing SNPs one by one can not perceive risk factors when the effect depends on the co-existence of two or more genes. The regulation of *HLA* and *KIR* on NK cell activation depends on each other through ligand-receptor interaction. In our study, *HLA-C1C1* and *HLA-Bw4-80I* could be detected as risk factors probably due to the nearly 100% presence of their *KIR* receptors in our population (table S1). However, *KIR* frequencies have been reported to be varied with geographical position and ethnic groups throughout China. For example, in Guangdong province, which is adjacent to Guangxi province where the GWAS is conducted, only 58% of the population possess *KIR2DL3* [41]. For *KIR2DS4/1D*, the susceptive effect depends on the presence of both *KIR2DS4* and *1D* as shown in table 2, and this effect could not be detected by GWAS neither. Hence, our association study, which investigated the combined effects of functional related polymorphic loci, could discover risk factors that could not be detected by GWAS.

Although the exact role of HBV genotype in hepatocarcinogenesis remains to be clarified, some genotypes are thought to increase the risk of HCC, such as HBV genotype C in Asian cohorts [42] or genotype F in other populations [43]. One limitation of our study is lack of information of HBV genotypes in the patients. Another limitation is that *KIR* allele polymorphisms were not analyzed in this study, since *KIR* polymorphisms, especially *KIR3DL1* polymorphisms had been reported to affect NK cell inhibition by *HLA* ligand [44]. Large prospective studies are needed to confirm the results of our study.

In conclusion, the results of the current study showed that HBV-infected patients with certain *KIR* and *HLA* genotypes were more likely to develop liver cancer, providing genetic evidence of that over-activation of NK cell might contribute to hepatitis B progressing to HCC development. Our study is useful for HCC surveillance, and has implication for personalized hepatitis B treatment aiming at HCC prevention.

## Supporting Information

Table S1 *KIR* frequencies in non-HCC and HCC patients.
(DOC)

Table S2 Associations of *HLA-C* genotypes with disease progression towards HCC.
(DOC)

Table S3 Combined effect of *HLA-Bw4-80I*, *HLA-C1C1*, and *KIR2DS4/1D* on HCC incidence.
(DOC)
