# Supplementary material for: KIR and HLA Loci Are Associated with Hepatocellular Carcinoma Development in Patients with Hepatitis B Virus Infection: A Case-Control Study
Source: PLoS One. 2011 Oct 5;6(10):e25682. doi: 10.1371/journal.pone.0025682 (PMC3187788; doi:10.1371/journal.pone.0025682)
Supplement: Table S1 — KIR frequencies in non-HCC and HCC patients. (DOC) [file pone.0025682.s001.doc]

Table S1. *KIR* frequencies in non-HCC and HCC patients

| *KIR* loci | Non-HCC n = 189 | HCC n = 144 | *p* | OR (95% CI)^c^ |
| --- | --- | --- | --- | --- |
|  | n (%) | n (%) |  |  |
| *KIR2DL1* | 186 (98.5) | 142 (98.6) | 1.0* | 1.15 (0.19 to 6.95) |
| *KIR2DL2* | 38 (20.1) | 29 (20.3) | 0.99 | 1.00 (0.58 to 1.72) |
| *KIR2DL3* | 187 (99.0) | 142 (98.6) | 1.00* | 0.76 (0.11 to 5.46) |
| *KIR2DL5* | 73 (38.7) | 61 (42.6) | 0.49 | 1.17 (0.75 to 1.82) |
| *KIR3DL1* | 180 (95.3) | 137 (95.3) | 0.97 | 0.98 (0.36 to 2.69) |
| *KIR2DS1* | 66 (34.9) | 56 (38.8) | 0.46 | 1.19 (0.76 to 1.86) |
| *KIR2DS2* | 37 (19.8) | 29 (19.9) | 0.90 | 1.04 (0.60 to 1.78) |
| *KIR2DS3* | 32 (16.8) | 30 (21.1) | 0.37 | 1.29 (0.74 to 2.45) |
| *KIR2DS4* | 145 (76.7) | 118 (81.9) | 0.25 | 1.38 (0.80 to 2.37) |
| *KIR1D* | 55 (29.1) | 53 (36.8) | 0.14 | 1.42 (0.89 to 2.25) |
| *KIR2DS5* | 49 (25.8) | 39 (27.0) | 0.81 | 1.06 (0.65 to 1.73) |
| *KIR3DS1* | 69 (36.4) | 54 (37.8) | 0.85 | 1.04 (0.67 to 1.64) |

* determined by Fisher’s exact test
