# Supplementary material for: KIR and HLA Loci Are Associated with Hepatocellular Carcinoma Development in Patients with Hepatitis B Virus Infection: A Case-Control Study
Source: PLoS One. 2011 Oct 5;6(10):e25682. doi: 10.1371/journal.pone.0025682 (PMC3187788; doi:10.1371/journal.pone.0025682)
Supplement: Table S2 — Associations of HLA-C genotypes with disease progression towards HCC. (DOC) [file pone.0025682.s002.doc]

Table S2. Associations of *HLA-C* genotypes with disease progression towards HCC

| *HLA-C* | Hepatitis n = 100 | Cirrhosis n = 80 | HCC n = 129 | Cirrhosis vs Hepatitis | | HCC vs Cirrhosis | | HCC vs Hepatitis | |
| --- | --- | --- | --- | --- | --- | --- | --- | --- | --- |
|  | n (%) | n (%) | n (%) | OR 95% CI | *p* | OR 95%CI | *p* | OR 95% CI | *p* |
| *HLA-C2C2* | 7 | 2 | 4 | 0.34 | 0.19* | 1.25 | 0.80* | 0.43 | 0.18* |
|  | (7.0) | (2.5) | (3.1) | 0.07 to 1.69 |  | 0.22 to 6.98 |  | 0.12 to 1.50 |  |
| *HLA-C1C2* | 27 | 36 | 28 | 2.21 | 0.01 | 0.34 | 0.001† | 0.75 | 0.35 |
|  | (27.0) | (45.0) | (21.7) | 1.19 to 4.13 |  | 0.19 to 0.62 |  | 0.41 to 1.38 |  |
| *HLA-C1C1* | 66 | 42 | 97 | 0.57 | 0.07 | 2.74 | 0.001† | 1.56 | 0.13 |
|  | (66.0) | (52.5) | (75.2) | 0.31 to 1.04 |  | 1.52 to 4.97 |  | 0.88 to 2.78 |  |

* determined by Fisher’s exact test

† Of significant difference after correction (*p* < 0.006)
