# Supplementary material for: KIR and HLA Loci Are Associated with Hepatocellular Carcinoma Development in Patients with Hepatitis B Virus Infection: A Case-Control Study
Source: PLoS One. 2011 Oct 5;6(10):e25682. doi: 10.1371/journal.pone.0025682 (PMC3187788; doi:10.1371/journal.pone.0025682)
Supplement: Table S3 — Combined effect of HLA-Bw4-80I, HLA-C1C1, and KIR2DS4/1D on HCC incidence. (DOC) [file pone.0025682.s003.doc]

Table S3. Combined effect of *HLA-Bw4-80I*, *HLA-C1C1*, and *KIR2DS4/1D* on HCC incidence

| Genetic factor | Non-HCC n = 169 | HCC n = 124 | *p*-Value | OR (95% CI)^c^ |
| --- | --- | --- | --- | --- |
|  | n (%) | n (%) |  |  |
| *HLA-Bw4-80I* and *HLA-C1C1* |  |  |  |  |
| *HLA-C1C1-*/*Bw4-80I-* | 62 (35.8) | 23 (17.8) | - | 1.00 (reference) |
| *HLA-C1C1+*/*Bw4-80I -* | 81 (46.8) | 60 (46.5) | 0.02 | 2.00 (1.11 to 3.58) |
| *HLA-C1C1-*/*Bw4-80I+* | 10 (5.8) | 9 (7.0) | 0.24 | 1.89 (0.64 to 5.55) |
| *HLA-C1C1+*/*Bw4-80I+* | 20 (11.6) | 37 (28.7) | 7.6E-06 | 4.99(2.42 to 10.29) |
|  | | | | |
| *HLA-C1C1, Bw4-80I* and *(2DS4/1D)* * |  |  |  |  |
| *HLA-C1C1/Bw4-80I*/*(2DS4/1D)-* | 52 (30.8) | 20 (16.1) | - | 1.00 (reference) |
| *HLA-C1C1-/Bw4-80I+ or (2DS4/1D)+* | 17 | 10 | 0.37 | 1.53 (0.60 to 3.90) |
| *HLA-C1C1+/Bw4-80I-*/*(2DS4/1D)-* | 65 (38.5) | 46 (37.1) | 0.07 | 1.78 (0.96 to 3.29) |
| *HLA-C1C1+/Bw4-80I-*/*(2DS4/1D)+* | 14 (8.3) | 13 (10.5) | 0.06 | 2.41 (0.97 to 6.02) |
| *HLA-C1C1+/Bw4-80I+*/*(2DS4/1D)-* | 16 (9.5) | 20 (16.1) | 0.005 | 3.25 (1.41 to 7.50) |
| *HLA-C1C1+/Bw4-80I+*/*(2DS4/1D)+* | 4 (2.4) | 13 (10.5) | 1.9E-04 | 8.45 (2.46 to 29.01) |
| *P* (trend test) = 7.4E-05 | | | | |

* *C1C1-/Bw4-80+/ (2DS4/1D)+* was not included because only 2 subjects in HCC group and 1 subject in control group carried this genotype
